# Measuring psychological safety in healthcare teams: developing an observational measure to complement survey methods

**DOI:** 10.1186/s12874-020-01066-z

**Published:** 2020-07-29

**Authors:** Róisín O’Donovan, Desirée Van Dun, Eilish McAuliffe

**Affiliations:** 1 Centre for Interdisciplinary Research, Education, and Innovation in Health Systems (IRIS), UCD Health Sciences Centre, School of Nursing, Midwifery & Health Systems, Health Sciences Centre, University College Dublin, Belfield, Dublin 4, Ireland; 2 Faculty of Behavioural, Management and Social Science, University of Twente, Enschede, The Netherlands

**Keywords:** Psychological safety, Healthcare teams, Measurement, Observation, Survey

## Abstract

**Background:**

Psychological safety is a dynamic team-level phenomenon which exists when team members believe that it is safe to take interpersonal risks. In healthcare teams, the presence of psychological safety is critical to delivering safe care. Scholars have highlighted a need for alternative measures which compliment survey-based measures of psychological safety in healthcare teams.

**Methods:**

The exploratory phase of this study raised concerns about whether current survey measures could provide a sufficient understanding of psychological safety within healthcare teams to inform strategies to improve it. Thus, previously validated psychological safety surveys and a meeting observation measure were adapted for use in healthcare teams. First, two group feedback sessions were held with 22 healthcare professionals, as well as a systematic literature review. Then, the members of eleven healthcare teams in Ireland and The Netherlands (*n* = 135) took part in the pilot test of the adapted composite measure.

**Results:**

The final composite measure has two parts: a team meeting observation measure and an adapted survey. The observation measure has 31 observable behaviours fitting seven categories: voice, defensive voice, silence behaviours, supportive, unsupportive, learning or improvement-oriented and familiarity type behaviours. The survey part consists of 19 items in three sub-dimensions related to; the team leader, other team members and the team as a whole. Three additional items capture the perceived representativeness of the observed team meeting compared to other similar meetings. Final adaptations were made in order to integrate the observation and survey measure.

**Conclusions:**

The resulting composite measure combines the strengths of observational and survey measures and is tailored for use in healthcare teams. It is uniquely co-developed with healthcare professionals and grounded in the psychological safety and healthcare literature. This composite measure can enable longitudinal research on psychological safety and inform future research to develop and test interventions to improve psychological safety.

## Background

Psychological safety is fundamental to effective teamwork, communication, and collaboration at work. It is “a team level phenomenon where all team members believe they are safe to take interpersonal risks” ([1], p. 354). Psychological safety is particularly relevant to healthcare teams because they work in a highly complex, dynamic, and high stakes work environment that requires them to work interdependently to co-ordinate safe patient care [2]. Healthcare teams need to be psychologically safe in order to maintain and encourage key outcomes, such as: patient safety [2–4], learning [1], and team performance [5]. Psychological safety also facilitates “teaming”, an active process which allows multidisciplinary healthcare teams to work together to deliver increasingly complex patient care [6, 7]. In psychologically safe team’s essential teamwork processes can evolve, including mutual performance monitoring, mutual trust, decision making, team cohesion, team motivation, and conflict resolution [8]. Moreover, how healthcare professionals relate to their co-workers is deemed an antecedent of job satisfaction [9]. To date, surveys have dominated the measurement of psychological safety [5]. Relying only on survey instruments can be burdensome for respondents and is limited by self-report bias [10–12]. Within a healthcare context, surveys are characterized by challenges such as respondent exhaustion and, consequently, low and declining response rates [13, 14]. This is likely to have worsened recently as the ongoing covid-19 pandemic has increased both physical and mental exhaustion among healthcare professionals [15, 16]. A recent paper by Mathieu and colleagues [17] calls for new methods of measurement which can complement surveys by providing additional insights into team dynamics. The observation measure presented as part of this paper addresses this need and allows for repeated tracking of healthcare team dynamics without requiring their time. Observation measures can complement surveys as they provide a more objective understanding, that is not hindered by low response or high attrition rates [12]. Being able to assess changes in psychological safety over time is particularly important as healthcare teams adapt to the challenges of the pandemic through developing new collaborative practices and evolving models of care delivery.

To date, empirical research has relied on cross-sectional, quantitative survey data to measure psychological safety [5]. Known survey measures of psychological safety ranged from three- to eight-item self-report scales [1, 2, 18–21]. While these scales have strong psychometric properties for measuring psychological safety and have been used within a healthcare setting, they were not developed within a healthcare setting. As a result, they may need to be adapted to fit different cultural and/or professional settings [22]. Healthcare settings are characterised by unique concerns related to patient safety and the increasing complexity of the care. These issues highlight the importance of having measures which can offer an in-depth understanding of psychological safety which is specific to healthcare settings [7, 23]. The current study presents the first survey measure of psychological safety which involved the target audience (healthcare professionals) in the process of adapting survey items. Previous studies have highlighted that involving end users in survey development can improve accuracy, quality and relevance of survey research [24]. To provide a more holistic understanding of psychological safety in healthcare teams, we also present an observational measure which complements the adapted survey. Relying purely on survey measures is a key shortcoming of organisational behaviour research [11]. Welp and Manser [25] found that survey studies which examine teamwork, clinician well-being and patient safety, have failed to take a holistic approach which accounts for the complexity of healthcare teams and have focused on the individual profession rather than the entire multi-professional team. Similarly, a recent systematic review of interventions aiming to improve psychological safety in healthcare teams, concluded that, in order to fully understand whether these interventions are successful, more holistic, objective measures are needed [26]. Observational techniques may offer insights into team psychological safety that the team themselves are not fully aware of and that complement findings from survey measures [5, 26, 27]. Recent literature reviews have highlighted the need for mixed-methods approaches to explore teamwork in healthcare and psychological safety [4, 26]. The complementary strengths of survey and observational methods of measurement can offer a more holistic understanding of psychological safety at both individual and team levels [28].

In this study, we respond to calls for improved, mixed method measures of psychological safety [4, 26]. Below we explicitly describe the methodology used to develop an observation measure that is grounded in the psychological safety and healthcare literature, to involve healthcare professionals in the adaptation of a psychological safety survey measure, and to pilot test both of these measures with healthcare teams.

## Methods

The present study aims to add to the existing body of literature by adapting current observational and survey measures, in collaboration with healthcare professionals, to provide a triangulated approach to measuring psychological safety at the team and individual level. For the purpose of this paper, a team will be defined as two or more healthcare professionals who: *“socially interact (face-to-face or, increasingly, virtually); possess one or more common goals; are brought together to perform organizationally relevant tasks; exhibit interdependencies with respect to workflow, goals, and outcomes; have different roles and responsibilities; and are together embedded in an encompassing organizational system, with boundaries and linkages to the broader system context and task environment”* ([26], p. 79). Team leaders will be included as part of the team because of the influence they have on psychological safety and collaboration within their teams [1–5, 29] this is especially the case in healthcare teams where team leaders typically function as actively involved foremen or -women [30].

### Exploratory phase

In the exploratory phase of this study, psychological safety was measured in an acute hospital in Ireland. While staff spoke about issues related to low psychological safety in their team or organisation, survey results did not reflect these issues. The full details of this exploratory phase can be found in Appendix A, which is included as a supplementary file. Feedback from participating healthcare professionals raised questions about the suitability of the survey questions for a healthcare context, as they experienced some difficulty in understanding and interpreting how the questions related to their context. In order to gain a more in-depth understanding of psychological safety within the case study hospital, the current study adapted the original survey items and developed a corresponding observation measure.

### Composite measure adaptation phase

**Fig. 1:**
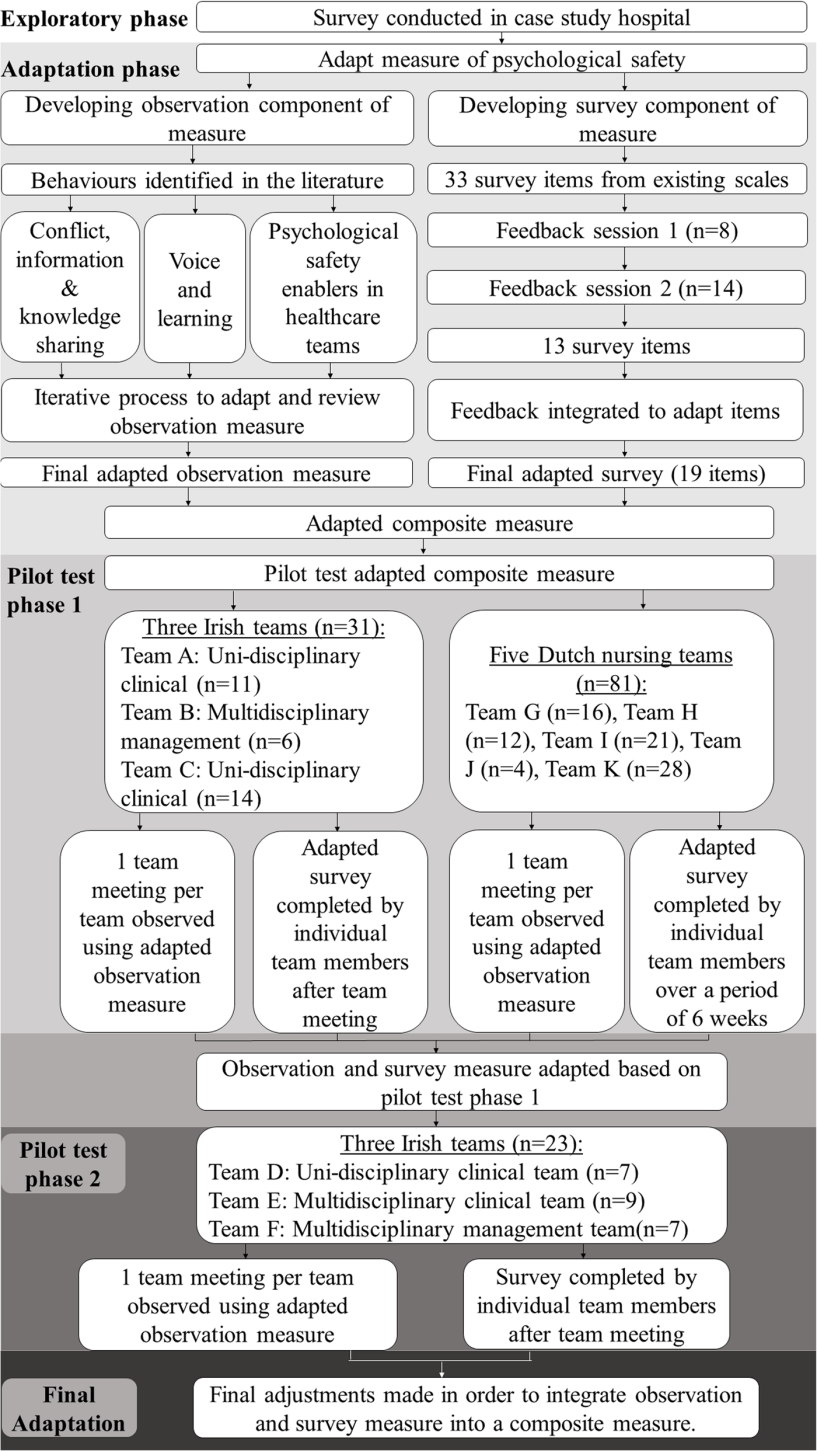
Process of Adapting and Pilot Testing the Composite Measure

Various steps were taken to develop a composite measure of psychological safety for use within healthcare teams. The composite measure consists of two parts: an observational measure and a survey measure. Both elements of the composite measure are adapted versions of previously used measures of psychological safety. Figure 1 illustrates the process through which the composite measure was developed, in two parallel streams.

#### Developing the observation measure component through reviewing the literature

The observation measure was developed based on behaviours which were identified from seminal research outlined below and are presented in Table 1.

**Table 1:** Behaviours Identified for Inclusion in Observation Measure

Source	Category	Behaviour
Hoenderdos [12]	1. Good Environment 2. Defensive or Evasive Behaviour 3. Responsibility 4. Feedback 5. Knowledge Sharing and Work Procedures 6. Extra behaviour Present in Meetings	1. Relaxed behaviour (make jokes, whistle, singing), Personal attention (talk about personal, non-work related matters), Enthusiasm (Greet, compliment), Agree (say yes, nod) 2. Aggression (raise voice, large gestures), Closed body posture (arms closed over each other, lean backwards), Evade confrontation (do not react to addressed problems or confrontations), Resistance against task (React negatively towards the execution of a task) 3. Point towards responsibility, Give fault to others (blame others as the source for own failure), Deny fault (deny any shortcoming of the alleged), Fail to meet or delay previous agreements (take responsibility and acknowledge own fault) 4. Active listening (Verify, confirm, paraphrase etc), Interrupting, Provide or ask for feedback, Do not make eye-contact during feedback. Joke about disagreements (about previous feedback, procedures or issues), React cold to enthusiasm. 5. Asking open questions, Ask or offer help, Brief consultation, Look for improvement opportunities (address own work method, look for solutions together), Discuss and compare results, Share procedures, knowledge and experiences, Negatively react towards ideas (ideas or opinions of other team members), Re-divide Tasks (of routine or previously divided tasks) 6. Not present of unprepared at the meeting (come late, read or work during meeting), Chatting or singing in sub-group, Give the same people the attention (with every new agenda topic)
Edmondson [1]	Learning behaviour	1. This team asks its internal customers for feedback on its performance 2. This team relies on outdated information or ideas 3. This team actively reviews its own progress and performance 4. This team does its work without stopping to consider all the information team members have 5. This team regularly takes time to figure out ways to improve its work performance 6. This team ignores feedback from others in the company 7. This team asks for help from others in the company when something comes up that the team members don’t know how to handle
Le Pine and Van Dyne [31]	Voice behaviour	1. Team member develop and make recommendations concerning issues that affect the work group 2. Team members speak up and encourage others in the group to get involved in issues that affect the group 3. Team members communicate opinions about work issues to others in the group even if they disagree 4. Team members keep well informed about issues where opinions might be useful to this work group 5. Team members get involved in issues that affect the quality of work life in the group 8. Team members speak up in the group with ideas for new projects of changes in procedures
Van Dyne et al. [32]	1. Acquiescent Silence 2. Acquiescent Voice 3. Defensive silence 4. Defensive voice 5. Prosocial Silence 6. Prosocial Voice	1. Remaining silent because of being disengaged or feeling unable to make a difference in the team. This may include; not speaking up or displaying disengaged non-verbal behaviour (e.g. no eye contact or closed body language) 2. Voicing passive support for ideas based on resignation or agree with the group due to low self-efficacy to make a difference. May include; expressing agreement without offering new ideas, not making effort to communicate own ideas. 3. Withholding information based on fear or self-protection. Not speaking up and displaying non-verbal behaviour which indicates fear (e.g. no eye contact, evades confrontation, closed body language) 4. Expressing ideas to shift attention away from self, based on fear. May include emphasising positive features and diverting attentions away from problems. 5. Withholding information based on cooperation or to benefit the team. Refusing to divulge information that might harm the organisation/team 6. Expressing solutions to problems based on cooperation or suggesting ideas for change to benefit the team. Speaking up about projects or ideas that might benefit the organisation and expressing ideas even if others disagree.
O’Donovan & McAuliffe [33]	1. Priority for safety 2. Improvement or Learning Orientation 3. Support 4. Familiarity with colleagues 5. Status, hierarchy and inclusiveness	1. *Safety Culture:* Conversations and discussion during meetings focused on safety. *Leader Behavioural Integrity for safety:* Team leaders words and actions aligned. *Professional Responsibility:* Individuals on the team refer to their responsibility for patients as a reason for disagreeing/speaking up. 2. *A Culture of Continuous Improvement:* The team is focused on continuous improvement- addresses areas related to improvement- asks questions related to improvement. *Change Oriented Leaders:* The team leaders behaviour shows that they encourage innovative thinking, change. The leader facilitates open discussion. 3. *Organisational Support:* Meetings are structured in a way that supports speaking up such as allowing time for questions and discussion. *Supportive Leadership:* Leader responds to employee input positively, gives freedom, autonomy, personal control to other team members, teaches and communicates strategic intent to their team, provides instruction, feedback and explanations about the procedure, makes sense of unexpected events and updates the status and progress of procedure. *Support from Peers:* In general, team members share decision making, speak up, show strong teamwork. 4. *Familiarity between Team Members:* Familiarity between team members evident- talking about personal issues, relaxed behaviour etc. *Familiarity across Teams:* Team members link information from other teams, suggest helpful collaboration with other teams and communicate with those outside the team to gain knowledge and expertise that they can report back to their group. *Familiarity with Leader:* Familiar relationships- talk personal, relaxed behaviour. 5. *Individual Knowledge and Experience:* Domination from one or a group of team members or sharing of power across team. *Leader Inclusivity:* Leader invites and appreciates input from other team members. Uses inclusive language such as “we”.

When developing an observation measure for team psychological safety, Hoenderdos [12] drew on *conflict and information or knowledge sharing* literature and identified 29 behaviours relevant to psychological safety (see Table 1). These were observed in 10 workplace teams. Because these teams were not from a healthcare setting, the observation measure requires further development and validation for use with healthcare teams. Since psychological safety may also be inferred from the extent to which people adopt *learning behaviours* within teams, the items developed by Edmondson [1] were included (see Table 1). These learning behaviours were identified during a study of teams in a manufacturing company. *Voice behaviours* were also included, based on the work conducted by Le Pine and Van Dyne [31] and Van Dyne et al. [32] (see Table 1). The framework proposed by Van Dyne et al. [32] is particularly helpful because it provides examples and definitions of behaviours associated with different types of employee silence and voice.

To ensure that the observation measure was directly relevant to healthcare teams, behaviours were identified in a systematic review of *enablers of psychological safety* within a healthcare context [33] (see Table 1). These behaviours were integrated into the observation measure to ensure it was suitable for use in a healthcare setting.

The behaviours presented in the above literature generated a long list of potential items to be included in the observation measure. Draft versions of the observation measure were reviewed and refined by the authors to ensure that each behaviour was observable and relevant to measuring psychological safety. As a criterion for inclusion the authors adhered to Bergner’s ([34], p. 147) definition, “*behavior i*s *any observable overt movement of the organism generally taken to include verbal behavior as well as physical movements*”. To ensure that healthcare staff could conduct routine, naked-eye observation assessments, [35], the observation measure had to fit on a single page and be easy to understand and use in the field.

#### Adapting the survey component through feedback sessions with healthcare professionals

Firstly, the survey component of the composite measure was adapted in consultation with healthcare professionals. Involving service users in healthcare research can identify research priorities, frame more rigorous research questions and produce higher levels of participation [36]. We conducted feedback sessions to adapt previously used psychological safety scale items for use in a healthcare context. The items which were adapted are presented in Table 2.

**Table 2:** Prominent Survey Measures of Psychological Safety

Authors	Items (Corresponding item in adapted survey)	Main features
Anderson & West [18]	1. We share information generally in the team rather than keeping it to ourselves (Q2, Q3, Q4, Q5, Q6, Q11, Q12, Q13, Q14, Q15, Q18, Q19) 2. We have a “we are together” attitude (Q9, Q10, Q17, Q18, Q19) 3. We all influence each other 4. *People keep each other informed about work-related issues in the team*^*b*^ (Q2, Q4, Q5, Q6, Q11, Q13, Q14, Q15, Q18, Q19) 5. People feel understood and accepted by each other (Q3, Q7, Q12, Q16) 6. Everyone’s view is listened to, even if it is in a minority (Q2, Q4, Q7, Q11, Q13, Q16) 7. *There are real attempts to share information throughout the team*^*b*^ (Q2, Q5, Q6, Q11, Q14, Q15, Q18, Q19) 8. There is a lot of give and take (Q17)	− Measures participative safety including: influence over decision making, information sharing, interaction frequency and safety. − Versions have been used in healthcare setting.
Edmondson [1]	1. If you make a mistake on this team, it is often held against you (Q5, Q6, Q14, Q15) 2. Members of this team are able to bring up problems and tough issues (Q1, Q2, Q3, Q5, Q6, Q10, Q11, Q12, Q14, Q15) 3. *People on this team sometimes reject others for being different*^*b*^ (Q4, Q7, Q13, Q16) 4. It is safe to take a risk on this team (All) 5. *It is difficult to ask other members of this team for help*^*b*^ (Q1, Q10, Q17) 6. No one on this team would deliberately act in a way that undermines my efforts (Q7, Q16) 7. *Working with members of this team, my unique skills and talents are valued and utilized*^*b*^ (Q4, Q7, Q8, Q13, Q16) 8. No one on this team would deliberately act in a way that undermines my efforts 9. *Working with members of this team, my unique skills and talents are valued and utilized*^*b*^	− Most commonly used measure of team psychological safety. − Developed through interviews and observations conducted within manufacturing company. − Has demonstrated good psychometric properties in various settings, including healthcare.
Edmondson & Wooley [19]	1. If I make a mistake in this job, it is often held against me (Q5, Q14) 2. It is difficult to ask others in this department for help^a^ (Q1, Q10, Q17) 3. *My manager often encourages me to take on new tasks or to learn how to do things I have never done before*^*b*^ (Q4, Q8) 4. If I was thinking about leaving this company to pursue a better job elsewhere, I would talk to my manager about it (Q2, Q9) 5. *If I had a problem in this company, I could depend on my manager to be my advocate*^*b*^ (Q2, Q3, Q9) 6. Often when I raise a problem with my manager, s/he does not seem very interested in helping me find a solution^a^ (Q1, Q2, Q3, Q5, Q6, Q9)	− Includes questions examining perceptions of managers and leaders regarding their support for speaking up.
Nembhard & Edmondson [2]	1. If you make a mistake in this team, it tends to be held against you^a^ (Q5, Q6, Q14, Q15) 2. *People in this unit are comfortable checking with each other if they have questions about the right way to do something*^*b*^ (Q1, Q2, Q10, Q11) 3. *The people in our team value others’ unique skills and talents*^*b*^ (Q7, Q8, Q13, Q16) 4. Members of this team are able to bring up problems and tough issues (Q1, Q2, Q5, Q6, Q11, Q12)	− Four items adapted from Edmondson (1999) scale to assess whether staff in a Neo-natal Intensive Care Unit felt psychologically safe.
Detert & Burris [20]	1. It is safe for me to make suggestions (Q4, Q7, Q13, Q16) 2. *It is safe for me to give my opinion*^*b*^ (Q2, Q4, Q7, Q11, Q13, Q16) 3. *It is safe for me to speak up around here*^*b*^ (Q1, Q2, Q3, Q4, Q5, Q6, Q7, Q10, Q11, Q12, Q13, Q14, Q15, Q16, Q17, Q18, Q19)	− Adapted Edmondson (1999)‘s team level items to capture individual level assessment of psychological safety within a restraint chain.
Garvin, Edmondson & Gino [21]	1. *In this unit, it is easy to speak up about what is on your mind*^*b*^ (Q1, Q2, Q3, Q4, Q5, Q6, Q7, Q10, Q11, Q12, Q13, Q14, Q15, Q16, Q17, Q18, Q19) 2. If you make a mistake in this unit, it is often held against you^a^ (Q5, Q6, Q14, Q15) 3. *People in this unit are usually comfortable talking about problems and disagreements*^*b*^ (Q2, Q3, Q5, Q6, Q9, Q11, Q14, Q15) 4. People in this unit are eager to share information about what does and doesn’t work (Q2, Q4, Q11, Q13, Q18, Q19) 5. Keeping your cards close to your chest is the best way to get ahead in this unit^a^ (All)	− Captures unit-level psychological safety. − Previously used to determine if a company functions as a learning organisation.

^a^Reverse scored

^b^ Items marked in italics were retained and presented at the second feedback session

Two group feedback sessions were conducted with 22 healthcare staff, working in Irish hospitals (session 1, *n* = 8; session 2, *n* = 14). All participants worked within different teams and were from different disciplines, including, nurses, consultants and physiotherapists. Given that the focus of these sessions was on gaining feedback on the survey items, no further specific demographic details were collected from participants. The first session lasted 40 min and the second session lasted 20 min. The process for conducting these feedback sessions is presented in Table 3.

**Table 3:** Process for Conducting Feedback Sessions to Adapt Survey Measures of Psychological Safety

Stage	Explanation	Output
**Workshop**		
Introduction	Researcher introduced the concept of psychological safety and gave examples relevant to healthcare settings	Healthcare professionals gained an understanding of psychological safety and the role it plays in teams.
Measurement development	Each participant was given items from six previously validated psychological safety scales (see Table 1). Each item was printed on individual slips of paper. Participants were invited to build their own measure of psychological safety by choosing the items they felt, based on their experience of working on healthcare teams, were most relevant. They could make any changes to the wording of items and add any relevant questions they felt were missing.	Each participant developed their own scale made up of the items they felt where most relevant to understanding psychological safety in healthcare teams.
Group discussion	The scales they developed were collected and there was a group discussion.	Feedback from the group, along with the items they chose to include in their scales were recorded and used to inform the adaptation of the survey.
**Post-workshop**		
Scale adaptation	Items from the original psychological safety scales were adapted based on feedback sessions	An initial draft of the adapted survey.
Discuss with research advisory panel	Draft versions of the scale were presented to and discussed with a research advisory panel made up of three academic researchers in the field of psychological safety and organisational change in healthcare settings.	Further iterations of the adapted survey.
Check-up with literature	The final scale was also reviewed to ensure the new items were in line with theoretical definitions of psychological safety from the literature (4,5).	Adapted survey to be used in the pilot tests (see Table 5).

### Pilot test phase

A pilot test was conducted to finalise the adapted composite measure. This pilot test was conducted within both Irish and Dutch hospitals. In Ireland, six hospital teams, ranging from management to clinical teams, took part and in the Netherlands, five nursing teams took part. The demographics of these teams are presented in Table 4. The teams’ weekly or monthly team meetings were observed, and team members completed the adapted survey. The pilot test was conducted over two phases (see Fig. 1). Three Irish and five Dutch teams took part in phase one. In phase two, the composite measure was updated based on the results from phase one and was then pilot tested with three other Irish teams.

**Table 4:** Participants in Pilot Test Demographics

Category	Irish Sample	Dutch Sample
Team size	Team A: *n* = 11 Team B: *n* = 6 Team C *n* = 14 Team D: *n* = 7 Team E: *n* = 9 Team F: *n* = 7	Team G: *n* = 16 Team H: *n* = 12 Team I: *n* = 21 Team J: *n* = 4 Team K: *n* = 28
Disciplines	Team A: Physiotherapy Team B: Multidisciplinary managers Team C: Nursing Team D: Speech and language therapy Team E: Multidisciplinary managers Team F: Multidisciplinary clinical^a^	All teams: Nursing
Gender	Female (*n* = 49) Male (*n* = 5)	Female (*n* = 78) Male (*n* = 3)
Total number participants	54	81

^a^Multidisciplinary clinical team members included: administrative staff, occupational therapists, social workers, psychologists, and dieticians

## Results

### Composite measure adaptation phase results

#### The adapted observation measure

Thirty behaviours were identified in the literature on conflict, information and knowledge sharing learning and voice behaviours and enablers of psychological safety in healthcare teams. These behaviours were grouped according to the following categories: Voice behaviours, defensive voice behaviours, silence behaviours, supportive behaviours, unsupportive behaviours, learning or improvement-oriented behaviours, familiarity behaviours and safety oriented behaviour (see Appendix B in supplementary files). Users of the observation measure can track the behaviours being observed by making the appropriate mark in the “behaviour count” box. Team members and team leaders’ behaviours are placed in separate columns in order to account for differences in psychological safety according to status or hierarchy and to observe whether leadership behaviours are influencing levels of psychological safety in the team [3, 5]. The observation measure also includes a section for capturing observers’ overall ratings after the meeting.

#### The adapted survey measure

Following the first feedback sessions, the items which at least 50% of the participants chose to include in their survey were identified. This was done in order to reduce the volume of items to be reviewed at the second session. Thirteen items were retained (see Table 2) and were given to the second group of healthcare professionals.

A second feedback session was conducted to check if other healthcare professionals agreed with the 13 items chosen by the first group. More than 50% of the participants chose to include all 13 survey items presented in the second feedback session (at least 64.3% of participants agreed on retaining each item). Only one reverse scored item was included in the final set of items. This was done in order to remain consistent with feedback from healthcare professionals, to keep items as close as possible to their original wording and to reduce any negative impact on reliability and validity [37].

Comments from both sessions were incorporated into the adapted set of survey items. The following section presents the main topics discussed during the feedback sessions. Overall, the participants felt that the original scale items did not capture enough detail to accurately measure psychological safety within healthcare teams. In particular, three key points were raised by them: the sensitivity of certain words in healthcare settings, the importance of the target of voice, and the difference between speaking up about personal or work issues.

First of all, the word “risk” was identified as a misleading word to use within a healthcare setting because it implies risky behaviour that could harm patients. The word “risk” appears in one of the items developed by Edmondson [1]; “It is safe to take a risk on this team”. Participants suggested that this item could be broken down into specific interpersonal risks, such as making suggestions for change, asking questions or reporting mistakes. This would avoid the use of the word “risk” and would give more detail of the kinds of interpersonal risks respondents feel safe taking.

Moreover, participants noted that it can be more difficult to engage in speaking up behaviour when it is directed at a superior, rather than team members who they consider to be their peers. Therefore, they suggested that items referring to feeling safe to voice opinions should be split into whether the respondent is voicing their opinion to their peers or to a superior. In line with this suggestion, questions referring to speaking up specified whether it was to a peer or a team leader.

Finally, healthcare professionals said that they would feel differently depending on whether they needed to speak up about a personal issue or a work issue. Based on their discussion, they suggested that questions specify whether people feel safe speaking up about a) personal issues and b) work issues.

The final list of items incorporated this feedback and can be seen in Table 5. This adapted scale is an extended version of the six previously published and validated psychological safety scales presented in Table 2. The items from these six scales are still present in our adapted version. The main difference between the adapted psychological safety scale and the original scales is that items are split into three sub scales: 1) questions related to psychological safety in relation to the team leader; 2) questions related to psychological safety in relation to peers/other team members of the team; and 3) questions related to psychological safety in relation to the team as a whole.

**Table 5:** Final Survey Items

Length of time working with this team: Please respond to the following questions by indicating your response between 1 = strongly disagree and 10 = strongly agree								
**Section 1.** Please answer the following questions in relation to your **team leader**								
Questions		^*b*^*1 Strongly disagree*	*2*	*3*	*4*	*5*	*6*	*7 Strongly agree*
1. If I had a question or was unsure of something in relation to my role at work, I could ask my team leader								
2. I can communicate my opinions about work issues with my team leader								
3. I can speak up about personal problems or disagreements to my team leader								
4. I can speak up with recommendations/ideas for new projects or changes in procedures to my team leader								
5. If I made a mistake on this team, I would feel safe speaking up to my team leader								
6. If I saw a colleague making a mistake, I would feel safe speaking up to my team leader								
7. If I speak up/voice my opinion, I know that my input is valued by my team leader								
8. My team leader encourages and supports me to take on new tasks or to learn how to do things I have never done before.								
9. If I had a problem in this company, I could depend on my team leader to be my advocate								
**Section 2.** Please answer the following questions in relation to your **peers/the other members of your team**								
Questions		^*b*^*1 Strongly disagree*	*2*	*3*	*4*	*5*	*6*	*7 Strongly agree*
10. If I had a question or was unsure of something in relation to my role at work, I could ask my peers								
11. I can communicate my opinions about work issues with my peers								
12. I can speak up about personal issues to my peers								
13. I can speak up with recommendations/ideas for new projects or changes in procedures to my peers								
14. If I made a mistake on this team, I would feel safe speaking up to my peers								
15. If I saw a colleague making a mistake, I would feel safe speaking up to this colleague								
16. If I speak up/voice my opinion, I know that my input is valued by my peers								
**Section 3.** Please answer in relation to your **team as a whole**								
Questions		^*b*^*1 Strongly disagree*	*2*	*3*	*4*	*5*	*6*	*7 Strongly agree*
17. *It is easy to ask other members of this team for help*^*a*^								
18. People keep each other informed about work-related issues in the team								
19. There are real attempts to share information throughout the team								
*Compared to similar meetings with your team how****different****was:*^*b*^								
*Questions*	*Very different*	*Different*	*Slightly Different*	*Neutral*	*Slightly different*	*Not different*		*Not at all Different*
20. *This meeting?*								
21. *Your behaviour during this meeting*								
22. *The behaviour of your colleagues*								

^a^Reverse scored

^b^The sections of the survey marked in italics were changed based on pilot tests

### Pilot test phase results

An iterative approach was taken to pilot testing the adapted composite measure (see Fig. 1). Based on these experiences and initial reliability testing of the adapted survey scales the following changes were made to the original measures.

#### Changes made to the original observation measure (see Appendix B in supplementary files)

- Behaviours which were observed during the team meetings and were not captured by the observation measure were added. These were: Correcting others, sharing future plans, acknowledging achievements/congratulating and delegating tasks. The behaviour “no eye contact (with speaker)” was deleted as it was already captured under “facial expressions indicate disengagement”.
- The behaviour “leaders’ words and deeds align” was deleted as it was not possible to observe this during team meetings.
- The category of behaviours originally labelled “safety-oriented behaviour” was removed and the remaining behaviour in that category, “informing the team about issues or mistakes related to patient safety” was moved to the category “Learning or improvement-oriented behaviour”.
- A section was added to record the duration of the observed meeting. This was done in order to allow future studies to standardize observations and compared them across teams.
- Clear definitions of each behaviour in the observation were decided upon (see Appendix C in supplementary files).
- The final observation measure had 31 items which were grouped according to the following categories: voice behaviours, defensive voice behaviours, silence behaviours, supportive behaviours, unsupportive behaviours, learning or improvement-oriented behaviours and familiarity behaviours.

#### Changes made to the survey measure (see Table 5)

The reliability outcomes from the teams which took part in pilot tests are presented in Table 6.

**Table 6:** Survey-based Reliability Outcomes of the Pilot Test

Variable	Team	α	Mean	s.d.
Psychological safety in relation to team leader (section 1)	Irish A-C	.874	6.729	.347
	Dutch G-K^a^	.953	5.435	.899
	Irish D-F^a^	.906	6.067	.834
Psychological safety in relation to peers (section 2)	Irish A-C	.878	6.314	.557
	Dutch G-K^a^	.955	5.475	.789
	Irish D-F^a^	.885	6.024	.834
Psychological safety in relation to the team as a whole (section 3)	Irish A-C	.377	5.778	.868
	Dutch G-K^a^	.637	5.166	.897
	Irish D-F^a^	.497	5.644	1.108
Full Scale	Irish A-C	.872	6.435	.414
	Dutch G-K^a^	.922	5.406	.660
	Irish D-F^a^	.956	5.989	.866
Meeting Representativeness	Irish A-C	NA	NA	NA
	Dutch G-K	NA	NA	NA
	Irish D-F	.916	6.079	1.037

^a^Survey results for Irish teams D-F and Dutch teams G-K were rescaled to a 1–7 scale for this analysis, in order to make all teams comparable

Based on these pilot outcomes, the survey part of the measure was adapted in the following ways:

- For Irish teams D-F and Dutch teams G-K, the original Likert scale of 1–7 was replaced by a scale of 1–10, with one indicating strongly disagree and 10 indicating strongly agree. The idea was to give participants an even wider spectrum of response options which allows the survey to capture more variability in participants’ responses [38]. The pilot tests conducted with Irish teams A-C used a 7-point Likert scale. Moving to a 10-point scale did not improve reliability outcomes, compared to the teams which used a 7-point scale. When compared to 7-point scales, 10-point scales have shown no marked difference in variance but have shown slightly lower mean scores [39]. Lastly, using a 7-point Likert scale facilitates easier comparison between the adapted survey and the original psychological safety surveys. Therefore, the adapted survey reverted to the 1–7 Likert scale.
- Section 3 of the survey, referring to the team as a whole, had a Cronbach’s alpha which was below 0.7 for both Dutch and Irish teams (see Table 6). The low Cronbach’s alpha for this section can be explained by the fact that it had only three items [40, 41]. In addition, this was the only section which included a reverse scored item, which may have negatively impacted reliability [37]. The alpha if item deleted score revealed that the Cronbach’s alpha could be improved by removing the reverse scored item (question 17). If question 17 was removed the Cronbach’s alpha for section 3 would have increased to .675 for Irish teams A-C (full scale α: .902), .791 for Dutch teams G-K (full scale α: .930) and .902 for Irish teams D-F (full scale α: .960). Therefore, it is recommended that this item should be changed to have the same polarity as the other items: “It is easy to ask other members of this team for help”.
- Three extra questions were added in order to capture participants’ perceptions of the representativeness of the observed meeting compared to other similar meetings [42]. Participants responses to these questions suggested that the meeting observed was not very different from other similar meetings. This outcome is also consistent with the fact that there was little reactivity to the researcher’s presence during the regular team meeting.

#### Integrating observation and survey measures to form a composite measure

A final adjustment was made to the observation measure in order to ensure that the observation and the survey measure could be integrated together, as well as to ease the analysis and comparison between the two. This final version of the observation measure included the target of each behaviour, specifically, whether the actor was directing their behaviour towards the team leader, other team members, or the team as a whole. In this version of the observation measure, there are two separate sections for recording team member behaviours and team leader behaviours. In addition, there is space for calculating the total score for both team leader and team members in categories that indicate a high score of psychological safety (voice behaviours, supportive behaviours, learning or improvement behaviour and familiarity behaviours) and the total score for the categories that relate to lower psychological safety (defensive voice behaviours, silence behaviours and unsupportive behaviours). This final observation measure is integrated with survey results and is presented in Table 7. Table 7 contains dummy data in order to visualise one of the various ways the data can be represented within the composite measure.

**Table 7:** Final Composite Measure Integrating Survey and Observation Components

Measurement	Psychological Safety Towards Team Leader		Psychological Safety Towards Other Team Members		Psychological Safety in Relation to Team as a Whole	
Survey Results	Mean	s.d.	Mean	s.d.	Mean	s.d.
	6.789	1.867	6.469	.986	5.987	.876
Total Observed Behaviours Displayed by:	Team Members		Team Leader	Team Members	Team Leader	Team Members
**Voice Behaviours**	**15**		**27**	**10**	**29**	**3**
Communicating opinions to others even if they disagree	2		5	2	5	
Asking questions	5		3	5	10	
Providing information	5		10		5	2
Providing feedback	3		3	2	6	1
Providing help or solutions			5		3	
Correcting others			2	1		
**Defensive Voice Behaviours**	**2**		**2**	**3**	**1**	
Denying faults or blame others				2		
Showing aggression				1		
Evading confrontation by focusing only on positives	2		2		1	
**Silence Behaviours**	**3**			**2**		**5**
Facial expression or body language indicates fear						
Facial expression or body language indicates disengagement	3					2
Closed body language				2		3
**Supportive Behaviours**	**10**		**19**	**7**	**17**	**1**
Sharing procedures, knowledge and experience	4		2		8	
Sharing future plans	2		3		2	
Active listening						
Use of inclusive language such as “we”			8		5	
Agreeing/Responding positively or enthusiastically to input	4			5		
Acknowledging achievements/ congratulating				2	2	1
Delegating tasks			6			
**Unsupportive Behaviours**			**3**	**4**		
Interrupting			3			
Discussions within small sub-groups				4		
Reacting cold/ignoring a joke						
**Learning or Improvement Oriented Behaviours**	**5**		**5**	**2**	**15**	**2**
Reviewing own progress and performance			2		7	
Asking for feedback	2					
Asking for help or solutions	1		3			
Asking for input from all meeting participants				2	8	1
Informing the team about issues or mistakes related to patient safety or staff safety						1
Looking for improvement opportunities and speaking up with ideas	2					
Acknowledging own mistake						
**Familiarity Behaviours**	**3**		**4**	**11**	**3**	**3**
Talking about personal, non-work matters			1	6		
Laughing about a joke	3		3	5	3	3
**Total Observed Behaviour**						
Categories indicating high psychological safety: (voice behaviours, supportive behaviours, learning or improvement behaviour and familiarity behaviours)	33		55	30	64	9
Categories indicating lower psychological safety: (defensive voice behaviours, silence behaviours and unsupportive behaviours).	5		5	9	1	5

### Interpretation and analysis

We designed the composite measure such that the data collected through the observation and survey methods can be triangulated during data analysis. To highlight the complementary components of the observation and survey measure, Table 8 presents the items which theoretically correspond to one another. In the final version of the composite measure, both the observation and survey data are split according to psychological safety related to; team leaders, other team members and the team as a whole. Presenting the results in this form facilitates comparison and triangulation of the results from both the observation and survey measures. While it would not be appropriate to combine the observation and survey results into one score, it is possible to triangulate both outputs to gain a more robust understanding of psychological safety within teams. For example, by comparing team members response to section 1 of the survey to the behaviours team members display towards their team leader during the observed meeting we can gain a better understanding of team members feelings of psychological safety in relation to their team leader. Triangulating the results from both measures can also facilitate exploration of whether team members feel more psychologically safe around their team leader or with other team members. We can better understand any differences in psychological safety by comparing the amount of voice behaviour team members direct towards other team members and their scores in the second section of the survey to the first section of the survey and the behaviour directed towards their team leader.

**Table 8:** Corresponding Observation and Survey Items

Behaviours	Corresponding survey questions
**Voice Behaviours**	
Communicating opinions to others even if they disagree	Q2, Q4, Q7, Q11, Q13, Q16, Q18, Q19
Asking questions	Q1, Q10, Q17
Providing information	Q2, Q3, Q4, Q5, Q6, Q11, Q12, Q13, Q14, Q15, Q18, Q19
Providing feedback	Q2, Q4, Q5, Q6, Q7, Q11, Q12, Q13, Q14, Q15, Q16, Q18, Q19
Providing help or solutions	Q4, Q13, Q18
Correcting others	Q15, Q18, Q19
**Defensive Voice Behaviours**	
Denying faults or blame others	Q5, Q14
Showing aggression (Raising voice, large gestures)	NA
Evading confrontation by focusing only on positives	Q5, Q6, Q14, Q15
**Silence Behaviours**	
Facial expression indicates fear	Q7, Q16
Facial expression indicates disengagement	Q4, Q7, Q8, Q13, Q16,
Closed body language (arms closed, lean backwards)	NA
**Supportive Behaviours**	
Sharing procedures, knowledge and experience	Q2, Q4, Q11, Q13 Q18, Q19
Sharing future plans	Q18, Q19
Active listening (verify, paraphrase)	Q1, Q10
Use of inclusive language such as “we”	NA
Agreeing/Responding positively or enthusiastically to input	Q7, Q16
Acknowledging achievements/ congratulating one another	Q8
Delegating tasks	Q8, Q17
**Unsupportive Behaviours**	
Interrupting	Q7, Q16
Discussions within small sub-groups	Q7, Q16
Reacting cold/ignoring a joke	NA
**Learning or Improvement Oriented Behaviours**	
Reviewing own progress and performance	Q1, Q4, Q10, Q13, Q19
Asking for feedback	Q1, Q10
Asking for help or solutions	Q1, Q10, Q17
Asking for input from all meeting participants	Q7, Q8, Q16
Informing the team about issues or mistakes related to patient safety	Q5, Q6, Q14, Q15, Q18, Q19
Looking for improvement opportunities and speaking up with ideas	Q4, Q8, Q13
Acknowledging own mistake	Q5, Q14
**Familiarity Behaviours**	
Talking about personal, non-work matters	Q12, Q3
Laughing about a joke	NA
**Overview Observations**	
There was enough opportunity for participants to ask for help	Q1, Q10, Q17
There was enough opportunity for participants to speak up	Q1, Q3, Q4, Q5, Q6, Q7, Q12, Q13, Q14, Q15, Q16, Q18, Q19
There was enough opportunity for participants to discuss with the team leader	Q1, Q2, Q3, Q4, Q5, Q6, Q7
Certain team members dominated the discussion	Q7, Q16
Decisions were made together, by the entire team	Q7, Q16
The atmosphere in this team was constructive	Q1, Q4, Q8, Q10, Q13, Q17, Q19
People seemed genuine and not to hold back anything	Q1, Q2, Q3, Q4, Q5, Q6, Q7, Q10, Q11, Q12, Q13, Q14, Q15, Q16, Q17, Q18, Q19

## Discussion

Previous studies identified the need to adapt and triangulate existing measures of psychological safety in healthcare teams to capture a more accurate and nuanced understanding of it at both individual and team levels [5, 26]. Building on previous research and feedback from healthcare professionals, this study describes the methodology used to adapt observational and survey measures of psychological safety, specifically for use within healthcare teams. The resulting adapted composite measure addresses concerns raised by healthcare professionals during the exploratory phase of this study, namely, that survey respondents may not have fully understood the questions, that the questions may not have been suitable for a healthcare context or that only the staff members with high psychological safety had responded to the survey. The adapted composite measure addresses these issues; since the survey items were adapted based on feedback from healthcare professionals, they are easily understood by healthcare professionals and directly relevant to a healthcare context. The observation measure provides a way to capture a team level measure of psychological safety that also includes staff members who do not complete the survey. In addition, the observation measure addresses calls for new methods of measurement that can complement survey measures and provide further insights into team dynamics, while minimising intrusiveness [17].

The similarities between the feedback from healthcare professionals and the psychological safety literature highlight the relevance and importance the adapted measure. The healthcare professionals highlighted a concern for patient safety, which has been found to facilitate psychological safety in healthcare teams [2, 3]. The importance of the target of voice was also highlighted as an issue. As noted by Amy Edmondson in her 2019 Academy of Management conference keynote speech, role-based status in teams may explain differences in team psychological safety. Healthcare professionals at the lower end of the hierarchy find it more difficult to speak to their superiors [2, 3]. Since the participating healthcare professionals recognised this, the resulting composite measure clarifies who participants feel comfortable speaking up to - their peers or their team leader.

The deliberate inclusion of common components across the survey and observation elements are a key strength of this adapted measure as it facilitates triangulation. The survey provides an understanding of individuals’ perceptions of psychological safety in relation to different organisational levels and regarding both professional and personal issues, while the observation measure targets observable behaviours associated with psychological safety at both the individual and team level. As previously mentioned, survey measures can be vulnerable to self-report bias, low response rates and are less suitable for longitudinal research due to response fatigue [11, 12, 14]. The observation measure compensates for these shortcomings by offering a more objective measure of psychological safety which can be repeated multiple times during longitudinal studies of psychological safety [12]. Given that psychological safety is seen as a state that can fluctuate over time, such longitudinal studies are called for [5]. The observation measure also offers an opportunity to capture data from employees who do not complete the survey and, as a result, reduce non-response bias. Using the observational and survey measures together also respects that the behaviour associated with silence and voice can contain elements of ambiguity [32], which make them challenging to interpret. To address this issue, the observation measure includes both verbal and non-verbal behaviours and individuals’ survey responses can further clarify any ambiguity in the observed behaviours. The results from both measures can be triangulated to understand the differences and similarities between self-reported levels of psychological safety and a more objective group level measure, thus providing a more robust and accurate assessment.

### Strengths, limitations and future research

A key strength of this study is that we have adapted and further developed existing measures of psychological safety rather than developing entirely new ones. The observation component is equally grounded in the healthcare and psychological safety literature and offers a dynamic, team-level measure of psychological safety. The survey component is based on valid and reliable survey items and has been adapted based on feedback from healthcare professionals to ensure it is tailored for healthcare settings. It is the first survey measure of psychological safety to have involved the target audience (healthcare professionals) in the developmental stage. This improves the accuracy, quality and relevance of the adapted survey [24, 36]. Although a limitation of this study is the relatively small number of healthcare professionals who participated in the feedback sessions, the same issues came up in both sessions, suggesting a sufficient level of data saturation. In addition, all members of the second feedback session agreed on the use of the scale items included in the final survey.

As well as explicitly describing the methodology used to adapt an observational and a survey measure of psychological safety, which is tailored for use within healthcare teams, this study pilot tests the resulting composite measure. This pilot test was conducted in both Dutch and Irish hospitals, offering preliminary evidence that the composite measure is suitable for use within different cultural contexts. In addition, the pilot test was conducted with a variety of healthcare team types, including uni-disciplinary nursing, physiotherapy and speech and language teams as well as multidisciplinary clinical and management teams. As a result, the composite measure is also suitable for use within a wide variety of healthcare team types. Given that this study covers the process of developing and pilot testing the adapted composite measure of psychological safety, future more rigorous testing with larger samples is necessary in order to examine its construct validity and reliability and further develop it. This future testing will enable the incorporation of feedback from a larger sample of healthcare professionals and will contribute to the development of normative data for the instrument. Further testing of the composite measure should also build on the theoretical link between the observation and survey items to test whether scores on the observation measure items are statistically correlated with behaviour counts for the corresponding survey items. Individual interviews with members of the healthcare teams are needed to gain information on individuals’ experiences on the team, their experience of completing the survey and of their team being observed. In subsequent stages we hope to observe regular team meetings with a video camera to enable the involvement of multiple independent raters, including the calculation of inter-rater reliability coefficients [42, 43].

The observational component of this measure will facilitate longitudinal research on psychological safety, which has been limited to date [5]. Longitudinally applying this new composite measure will allow researchers to gain a more dynamic and holistic perspective of psychological safety which will expand our knowledge of how psychological safety works within healthcare teams. This will address the call made by Welp and Manser [25], for measures of teamwork in healthcare to account for temporal instability of healthcare teams.

### Implications for practice

Healthcare teams can use this composite measure to learn about and improve psychological safety within their team. Harris et al. [27] suggest that it is feasible for healthcare professionals to undertake structured observational assessment and teams find feedback from such observations useful. We anticipate healthcare professionals using our observation measure to observe teams within their hospital to better understand psychological safety and stimulate team learning accordingly. It can also be used by team leaders or their coaches to identify observable behaviours related to psychological safety in their team. Being more conscious about such behaviours enables team leaders to find the root causes and act upon them. The outcomes of our work also inform Human Resource professionals. They can train team members to detect psychologically unsafe work situations and adopt relations-oriented behaviours that support a more psychologically safe, inclusive work climate instead [44].

Given the important role of psychological safety in healthcare teams, there is a need to understand the concept fully in order to consider how it can be improved [5, 26]. Creating and maintaining psychological safety will be paramount in dealing with the covid-19 crisis [45]. Healthcare teams will be required to draw on knowledge and learning from all parts of the healthcare system in order to make quick decisions, learn from mistakes and implement changes that will facilitate the safe delivery of care [45]. By gaining a better understanding of psychological safety in healthcare teams and using this knowledge to develop and implement interventions to improve psychological safety, we can increase team’s ability to learn, co-ordinate care and make decisions that will ultimately result in higher team performance. To date, there has been little guidance on how teams can introduce, improve, and maintain psychological safety. A recent systematic review identified both a lack of interventions targeting psychological safety and a lack of objective measures for identifying improvements in psychological safety [26]. The adapted measure presented in this article can be used to both inform the development of interventions to improve psychological safety, by gaining a more holistic understanding of it, and to assess the effectiveness of these interventions.

## Conclusions

This study describes the methodology used to adapt observational and survey measures of psychological safety for use within healthcare teams, by building upon the feedback retrieved from the target audience: healthcare professionals. It also reports on a pilot test of the resulting composite measure. The adapted composite measure combines the strengths of observational and survey measures in order to gain a more holistic, nuanced understanding of psychological safety within healthcare teams, enable longitudinal research on psychological safety and inform future research to develop and test interventions to improve psychological safety.

## Supplementary information

**Additional file 1: Appendix A.** Exploratory Phase Description. Table A1 Exploratory Phase Participant Information. **Appendix B.** Observation Measure Used During Pilot Tests. **Appendix C.** Definition of Behaviours in Observation Measure.

## Data Availability

The datasets used and/or analysed during the current study are available from the corresponding author on reasonable request.

## Abbreviation

IRIS: Centre for Interdisciplinary Research, Education, and Innovation in Health Systems
