# Supplementary material for: Measuring psychological safety in healthcare teams: developing an observational measure to complement survey methods
Source: BMC Med Res Methodol. 2020 Jul 29;20:203. doi: 10.1186/s12874-020-01066-z (PMC7387873; doi:10.1186/s12874-020-01066-z)
Supplement: Supplementary file 1 — Additional file 1: Appendix A. Exploratory Phase Description. Table A1 Exploratory Phase Participant Information. Appendix B. Observation Measure Used During Pilot Tests. Appendix C. Definition of Behaviours in Observation Measure. [file 12874_2020_1066_MOESM1_ESM.docx]

**Appendices**

**Appendix A**

**Exploratory Phase Description**

This hospital had 106 beds and approximately 400 staff members. Edmondson’s [1] seven-item measure of psychological safety in teams was used and proved to be reliable (α = .802). This survey measure was chosen as it is the most commonly used measure of psychological safety and has demonstrated good psychometric properties in a variety of samples [5]. The researcher who administered the survey had been observing team meetings and talking to staff in the hospital for eight months prior to the survey. Staff regularly spoke about issues related to low psychological safety in their team or organisation, which suggested that the survey results would also reflect low levels of psychological safety. Surprisingly, when the surveys were analysed, this was not the case. Of the 481 staff working at the hospital, 107 responded to the survey (22.24% response rate). Information on the participants can be found in Table A1. The majority of staff either agreed (49.5%, n = 53) or somewhat agreed (19.6%, n = 21) that their team was psychologically safe. Only 14 (13.1%) respondents reported that there was low psychological safety in their team (somewhat disagree n=13, disagree n=1). The mean score for total psychological safety was 5.41 and the standard deviation was 1.2. In contrast with previous research which suggests that team members with a higher status within the team will have higher psychological safety than those with lower status [2,4,5], a Kruskal-Wallis Test revealed no significant difference in psychological safety levels according to participants’ status within the team (i.e. whether they were entry, middle or senior grade).

**Table A1**

**Exploratory Phase Participant Information**

| Category | Respondents (percentage) |
| --- | --- |
| Grade/level in hospital |  |
| Entry  Middle  Senior  Not indicated | 23 (21.5%)  29 (27.1%)  17 (15.9%)  38 (35.5%) |
| Profession |  |
| Nurses  Physiotherapists  Administrative staff  Support staff  Medical staff  Occupational therapy  Speech and language therapy  Social work or psychology  Human Resources  Catering staff  Not indicated | 21 (19.6%)  10 (9.3%)  8 (7.5%)  8 (7.5%)  7 (6.5%)  5 (4.7%)  5 (4.7%)  4 (3.7%)  2 (1.9%)  2 (1.9%)  35 (32.7%) |
| Total number participants | 107 |

This exploratory study highlighted the need to gain a more in-depth understanding of psychological safety within the case study hospital. The anonymised results of the survey were presented to staff from the same hospital during a feedback session. Staff feedback on the results confirmed the researchers’ concerns that the scale was not accurately capturing the perceived and experienced levels of psychological safety in the hospital. The participating healthcare professionals suggested that survey respondents may not have fully understood the questions, that the questions may not have been suitable for a healthcare context or that only the staff members with high psychological safety had responded to the survey. Another interpretation may be that Edmondson’s [1] original scale, which had been developed and validated with a North-American sample, may need to be adapted to fit different cultural and/or professional settings [22].

**Appendix B**

**Observation Measure Used During Pilot Tests**

| **Behaviours** | **Team member** behaviour count* | **Team leader** behaviour count* |
| --- | --- | --- |
| **Voice Behaviours** |  |  |
| Communicating opinions to others even if they disagree |  |  |
| Asking questions |  |  |
| Providing information |  |  |
| Providing feedback |  |  |
| Providing help or solutions |  |  |
| *Correcting others*** |  |  |
| **Defensive Voice Behaviours** |  |  |
| Denying faults or blame others |  |  |
| Showing aggression (Raising voice, large gestures) |  |  |
| Evading confrontation by focusing only on positives |  |  |
| **Silence Behaviours** |  |  |
| Facial expression indicates fear |  |  |
| Facial expression indicates disengagement |  |  |
| Closed body language (arms closed, lean backwards) |  |  |
| ~~*No eye contact (with speaker)~~ |  |  |
| **Supportive Behaviours** |  |  |
| Sharing procedures, knowledge and experience |  |  |
| *Sharing future plans*** |  |  |
| Active listening (verify, paraphrase) |  |  |
| Use of inclusive language such as “we” |  |  |
| Agreeing/Responding positively or enthusiastically to input |  |  |
| *Acknowledging achievements/ congratulating one another*** |  |  |
| *Delegating tasks*** |  |  |
| **Unsupportive Behaviours** |  |  |
| Interrupting |  |  |
| Discussions within small sub-groups |  |  |
| Reacting cold/ignoring a joke |  |  |
| **Learning or Improvement Oriented Behaviours** |  |  |
| Reviewing own progress and performance |  |  |
| Asking for feedback |  |  |
| Asking for help or solutions |  |  |
| Asking for input from all meeting participants |  |  |
| *Informing the team about issues or mistakes related to patient safety*** |  |  |
| Looking for improvement opportunities and speaking up with ideas |  |  |
| Acknowledging own mistake |  |  |
| **Familiarity Behaviours** |  |  |
| Talking about personal, non-work matters (with team members) |  |  |
| Talking about personal, non-work matters (with team leader) |  |  |
| Laughing about a joke |  |  |
| **~~*Safety Oriented Behaviour~~** |  |  |
| ~~*Leaders’ words and deeds align~~ |  |  |
| ~~*Informing the team about issues or mistakes related to patient safety~~ |  |  |

Observer ratings after meeting: Rated from 1 (strongly disagree) to 7 (Strongly agree)

| Observations | 1 Strongly disagree | 2 | 3 | 4 | 5 | 6 | 7 Strongly agree |
| --- | --- | --- | --- | --- | --- | --- | --- |
| There was enough opportunity for participants to ask for help |  |  |  |  |  |  |  |
| There was enough opportunity for participants to speak up |  |  |  |  |  |  |  |
| There was enough opportunity for participants to discuss with the team leader |  |  |  |  |  |  |  |
| Certain team members dominated the discussion |  |  |  |  |  |  |  |
| Decisions were made together, by the entire team |  |  |  |  |  |  |  |
| The atmosphere in this team was constructive |  |  |  |  |  |  |  |
| People seemed genuine and not to hold back anything |  |  |  |  |  |  |  |

*Meeting duration:*

*These items have been crossed out as they were removed or moved to another category following the first three pilot tests.

**The items marked in italics were added following the first eight pilot tests.

**Appendix C**

**Definition of Behaviours in Observation Measure**

| **Behaviours** | **Definitions** |
| --- | --- |
| **Voice Behaviours** |  |
| Communicating opinions to others even if they disagree | Sharing opinions that contradict what others have said or that other team members disagree with. |
| Asking questions | Speaking up with any question. |
| Providing information | Providing others with relevant information, updating them on issues. |
| Providing feedback | (Constructively) Commenting on other’s input or behaviour |
| Providing help or solutions | Offering to take part in correcting a problem or issue or suggesting a solution. |
| Correcting others | Speaking up to correct what is being said by another team member. |
| **Defensive Voice Behaviours** |  |
| Denying faults or blame others | When discussing mistakes or problems, speaker denies own fault/blames others. |
| Showing aggression | Displays of aggression in voice or body language, i.e. raised voice, or large gestures. |
| Evading confrontation by focusing only on positives | When a difficult or confrontational issue is being discussed, the team member shifts focus to more positive issues rather than directly addressing the difficult issue. |
| **Silence Behaviours** |  |
| Facial expression or body language indicates fear | Any indication of fear (i.e. darting eye movements or nervous shifting in chair) either when speaking, during silence or while listening to others. |
| Facial expression or body language indicates disengagement | Any indication of disengagement (i.e. looking around the room, at phone or body facing away from others) either when speaking, during silence or while listening to others. |
| Closed body language | Team members arms are closed, they are leaning backwards. |
| **Supportive Behaviours** |  |
| Sharing procedures, knowledge and experience | Sharing a solutions or learning experiences contributing to teams understanding of the issue at hand and team learning. |
| Sharing future plans | Providing information on actions or procedures which will take place after meeting. |
| Active listening | Responding to what others are saying by paraphrasing or verifying what was said. |
| Use of inclusive language such as “we” | Speaker refers to the team using “we” rather than “I” and “you”. |
| Agreeing/Responding positively or enthusiastically to input | Positive response to others i.e. agreeing, nodding or reinforcing what has been said. |
| Acknowledging achievements/ congratulating | Team members opening acknowledge achievements and congratulate one another on them. |
| Delegating tasks | Tasks or responsibilities are shared across the team. |
| **Unsupportive Behaviours** |  |
| Interrupting | Team member talks over another or steers the discussion away to another topic. |
| Discussions within small sub-groups | A small number of team members have a sub-conversation during which they are temporarily disengaged form the team discussion and do not share with others. |
| Reacting cold/ignoring a joke | When a joke is made, the team member either does not laugh or continues as if they did not hear the joke. |
| **Learning or Improvement Oriented Behaviours** |  |
| Reviewing own progress and performance | Discussions on reviewing initiatives or aspects of performance that are working well or need to be improved. |
| Asking for feedback | Team member requests for input from other team members on what they have said. |
| Asking for help or solutions | Speaking up with requests for help or suggestion of solutions from other team members. |
| Asking for input from all meeting participants | An open request to all team members for input. |
| Informing the team about issues or mistakes related to patient safety or staff safety | Speaking up about and discussing issues that are directly relevant to patient or staff safety. |
| Looking for improvement opportunities and speaking up with ideas | Speaking up with suggestions for improvement in relation to issue of relevance to the team. |
| Acknowledging own mistake | Team member acknowledges a mistake they made. The team member does not blame others or circumstances and acknowledges own short coming. |
| **Familiarity Behaviours** |  |
| Talking about personal, non-work matters | Any discussion which is not about work |
| Laughing about a joke | Team laughs together at a joke or funny comment |
